# Differences between endogenous attention to spatial locations and sensory modalities

**DOI:** 10.1007/s00221-017-5030-4

**Published:** 2017-07-17

**Authors:** J. Vibell, C. Klinge, M. Zampini, A. C. Nobre, C. Spence

**Affiliations:** 1 Department of Experimental Psychology, University of Oxford, Oxford, UK; 2 Department of Psychology, University of Hawaii, 2530 Dole St, Honolulu, HI 96822 USA

**Keywords:** Prior entry, Temporal-order judgments, Event-related potentials, Attention, Crossmodal, Visual, Tactile, P1, N1, P2, P300

## Abstract

Vibell et al. (J Cogn Neurosci 19:109–120, 2007) reported that endogenously attending to a sensory modality (vision or touch) modulated perceptual processing, in part, by the relative speeding-up of neural activation (i.e., as a result of prior entry). However, it was unclear whether it was the fine temporal discrimination required by the temporal-order judgment task that was used, or rather, the type of attentional modulation (spatial locations or sensory modalities) that was responsible for the shift in latencies that they observed. The present study used a similar experimental design to evaluate whether spatial attention would also yield similar latency effects suggestive of prior entry in the early visual P1 potentials. Intriguingly, while the results demonstrate similar neural latency shifts attributable to spatial attention, they started at a somewhat later stage than seen in Vibell et al.’s study. These differences are consistent with different neural mechanisms underlying attention to a specific sensory modality versus to a spatial location.

## Introduction

Arguments concerning the law of prior entry, originally formulated by Titchener a little over a century ago (Titchener 1908), have, over the last 15 years or so, developed into a debate about whether or not attention speeds-up perceptual processing in comparison to relatively less attended (or unattended) stimuli. Spence et al. (2001) revived interest in this topic, highlighting a number of inconsistencies in earlier studies of prior entry. Subsequent behavioural studies have investigated a number of the finer mechanics of prior entry (e.g., see Lester et al. 2009; Matthews et al. 2016; McDonald et al. 2005; Miyazaki et al. 2016; Olivers et al. 2011; Schneider and Bavelier 2003; Spence and Parise 2010; Vibell et al. 2007; Weiss and Scharlau 2009, 2011, 2012; West et al. 2009; Yates et al. 2009, 2011; Zampini et al. 2005; Zhuang and Papathomas 2009).

In 2005, McDonald et al. (2005) investigated the neural underpinnings of the prior-entry effect using electrophysiological recordings. These researchers were interested in understanding the physiological mechanisms by which attention speeds-up perceptual processing. These researchers had their participants perform a temporal-order judgment (TOJ) task while event-related potentials (ERPs) were recorded. The TOJ task has, for many years, been a favoured method for evaluating prior entry amongst researchers. The task in the majority of studies typically involves participants making unspeeded perceptual judgments, thus allowing for the demonstration of genuinely perceptual modulations by attention. This stands in contrast to the more commonly reported reaction-time (RT) studies, in which the contribution of attentional modulations occurring at later cognitive and response-related stages are rather more difficult to eliminate (e.g., Watt 1991).

The focus of a participant’s attention was manipulated in McDonald et al.’s (2005) study by means of exogenous spatial cuing with an auditory noise burst, presented to the left or right shortly before the presentation of two visual stimuli, one to either side of central fixation. The visual stimuli were presented at a variety of stimulus onset asynchronies (SOAs). The results revealed that the point of subjective simultaneity (PSS) was modulated by spatial attention. The PSS, calculated from the pattern of performance at different SOAs, indicating the interval at which the left- and right-first responses would be made equally often. In this case, the left visual stimulus had to be presented relatively earlier in time when the spatially-non-predictive auditory cue was presented on the right than when it was presented on the left. Electrophysiological recordings revealed that this effect was indeed accompanied by a neural modulation taking place as early as the early extrastriate analysis of the visual stimuli, as reflected by the P1 potential (see also McDonald et al. 2013; Störmer et al. 2009). The underlying mechanism, however, demonstrated no significant speeding-up of the latency of the sensory brain potentials, but rather an increase in the amplitude of the early visual brain potentials. An enhanced positivity contralateral to the cued visual target starting in the P1 interval and lasting for ~100 ms appeared to lead to the perceived shifts in temporal order. McDonald et al. did not see a speeding-up of the neural processes underlying perceptual processing itself, as might have been inferred from the law of prior entry. That said, it is uncertain whether McDonald et al.’s results actually reflect an enhancement of perceptual processing by attention or a second-order response bias [note that the same argument can be applied to Vibell et al.’s (2007), study too], the early effect suggests that the effect is attentional as discussed in their study. In second-order response-biases participants simply use attentional instruction to guide their selection of appropriate response once the two stimuli have been presented. Santangelo and Spence (2008) argued that the TOJ effects that have been documented to date could be attributable to cue-induced response bias. However, the crossmodal cuing of visual stimuli ruled out that these effects could stem from lower level intramodal processes and suggest they occur by way of a supramodal attention system, or inter-modal connectivity, instead (Störmer et al. 2009).

The shift in amplitudes at the perceptual stages of information processing fits well with the extant literature on both endogenous and exogenous spatial attention. At these stages, spatial attention is thought to operate by means of sensory gating or gain modulation (Eimer 1994; Hillyard et al. 1998; McDonald et al. 2005; Näätänen 1986), whereby the processing of relevant, attended stimuli is boosted relative to the processing of irrelevant stimuli starting from early stages of information processing. Both the enhancement of neuronal activity related to attended stimuli and the attenuation of neuronal activity associated with ignored stimuli may contribute to sensory gating and gain control (Anllo-Vento et al. 2004; Eimer 1994; Hillyard et al. 1998; Mangun and Hillyard 1991; Tünnermann et al. 2015). Attentional modulation through gating or control of the amount of neural activity has been considered a ubiquitous mechanism, though it should be noted that support for such a claim has come primarily from those studies in which attention has been manipulated in the spatial domain (Hillyard et al. 1998).

Vibell et al. (2007) conducted an ERP study that highlighted the possibility that prior entry can be expressed during early perceptual processing, at least when a participant’s attention is endogenously directed to a specific sensory modality rather than to a particular spatial location. Using a crossmodal TOJ paradigm based on earlier psychophysical studies reported by Spence et al. (2001), the participants’ attention was directed to either vision or touch, while they responded orthogonally to which side stimuli appeared first. Latency shifts of visual potentials were observed, with processing occurring earlier when the visual stimuli were attended as compared to when they were relatively ignored. Latencies were shifted by 3–4 ms in terms of the perceptual P1 and N1 potentials and by 15 ms in the cognitive P3 potential. PSSs have been shown to correlate with the P1 and N1 latency (e.g., Boenke et al. 2012), but the latencies of early potentials do not appear to shift in line with attention. Comparing Vibell et al.’s results with those from McDonald et al. (2005) highlighted the possibility that the neural mechanism underlying prior entry might depend on the type of attention being manipulated (i.e., attention to a spatial versus attention to a sensory modality). They suggest that a somewhat different mechanism than sensory gating may be at work. The results of Vibell et al.’s study, therefore, raises the intriguing possibility that attention to a sensory modality and attention to a location in space may involve different underlying neural mechanisms, at least under those conditions in which participants have to make fine temporal discrimination responses, such as those required in TOJ tasks.

It is important to note, though, that a concern has been raised about the most appropriate interpretation of Vibell et al.’s (2007) findings by Keetels and Vroomen (2012, p. 152) and McDonald et al. (2012, p. 516). Keetels and Vroomen noted that “the 4-ms shift in the ERP is in a quite different order of magnitude than the 38-ms shift of the PSS in the behavioural data, or the 133-ms shift reported by Spence et al. (2001)”. This concern was a common concern addressed in the original paper (Vibell et al. 2007), where the reasons as to why latencies in ERP potentials are smaller than correlated shift in behavior were discussed^1^ (see also Tünnermann and Scharlau 2016).

Over the years, several researchers have tried to compare the effects of attention to spatial locations versus attention to sensory modalities on neural activity, but differences in experimental design make direct comparisons difficult (de Ruiter et al. 1998; Eimer and Schröger 1998; Hotting et al. 2003; Talsma and Kok 2001, 2002; Teder-Sälejärvi et al. 1999; Woods et al. 1992, 1993). Several of the experiments mentioned above (De Ruiter et al. 1998; Talsma and Kok 2001, 2002; Woods et al. 1992, 1993) failed to present the stimuli from different modalities from the same spatial location (e.g., auditory stimuli were typically presented over headphones, while the visual stimuli were presented on a computer screen). This is an important caveat, because it may potentially have biased the whole process of spatial attention between modalities (see Spence and Driver 1997; Spence et al. 2004, on this point). The different spatial locations might serve as additional cues for attention, so these results should be interpreted with some degree of caution. Meanwhile, the other studies (Hötting et al. 2003; Teder-Sälejärvi et al. 1999) all required their participants’ attention to be focused on a specific sensory modality at a specific location in the same way as in Eimer and Schröger’s (1998) earlier study.

Those studies that have controlled the locations from which the stimuli have been presented have not investigated the effects of attention to a modality in isolation from the effects of attention to spatial location. Eimer and Schröger (1998) tried to distinguish spatial from sensory attention by directing participants’ attention to sensory modalities and indicating spatial locations by a cue. Similar approaches have also been adopted by other researchers, where attention has been directed to non-spatial dimensions such as to the features and objects (Anllo-Vento and Hillyard 1996; Eimer 1997; Hillyard and Munte 1984; Hopf et al. 2004; Valdes-Sosa et al. 1998). Therefore, the possibility that attention to a sensory modality, if it can be separated from attention being directed to its intrinsic spatial location, might involve different neural mechanisms has not yet been satisfactorily explored. Indeed, McDonald et al. (2012, p. 516) point out that: “it is tempting to speculate that voluntary modality-based attentional selection influences the timing of early visual activity, whereas involuntary location-based attentional selection influences the gain of early visual activity”.

Another possible explanation for the latency shifts in perceptual potentials observed in the TOJ experiment reported by Vibell et al. (2007), when the attention of participants was directed to a sensory modality, was a combination of two factors. The requirement for participants to make perceptually difficult temporal discriminations in combination with a task that was sensitive to specific attentional manipulations could have caused the latency shifts. Earlier studies have shown ERP latency shifts when attention is oriented to a specific point in time using both peripheral (Griffin et al. 2002) and foveal stimuli (Miniussi et al. 1999). However, none of these previous studies revealed any effect on early perceptual analysis (P1) as a function of temporal attention. Instead, the modulation was of later potentials (P3) thought to be more involved in decisional and response-related processes. These previous temporal-orienting studies, however, did not require their participants to make any fine-grained temporal judgments. Hence, we thought it possible that it might be the combination of the strong perceptual demands during the TOJ task, its reliance on temporal processing, and the fact that attention was directed to a specific sensory modality that were together responsible for the early modulations of latencies observed by Vibell et al.

The early latency shifts reported by Vibell et al. (2007) might be attributable to the paradigm differing in a few other specific ways from the previous TOJ study reported by McDonald et al. (2005). First, attention was directed toward one sensory modality (touch or vision) on both sides of fixation (i.e., attention was divided spatially, or at least not focused on a specific spatial location). Directing attention to one sensory modality irrespective of which side the cues and target stimuli are presented from could have the advantage that the main focus of attention is on sensory perception per se (as opposed to its inherent spatial component) and not as much on the location that is inherently linked with the sensory perception. In McDonald et al.’s study, attention was directed by a non-predictive auditory cue, but it was presented at a specific location. In the current study and in Vibell et al. (2007), attention was directed to touch and vision irrespective of location. Second, attention was oriented in a sustained fashion by presenting a higher percentage of stimuli in the attended modality and by verbal instruction given by the experimenter. Prior work has shown more effective attentional orienting with sustained rather than transient attention, resulting in both peak latency shifts (Eimer and Forster 2003) amplitude modulations (see Eimer 1996).

The main aim of the present study was, therefore, to apply the same design as the study by Vibell et al. (2007) to test whether equivalent behavioural and neural prior-entry effects would occur with endogenous spatial attention. To maintain orthogonal attention and response dimensions, the participants responded to the modality of the target stimulus that occurred first, while attention was directed to spatial locations (either to the left or right). This design enabled us to compare results to our earlier findings using exactly the same stimulation parameters, but with spatial attention being manipulated instead of attention to a specific sensory modality. Of particular interest, here was the question of whether sustained spatial attention in the TOJ task would induce changes in relative latency or amplitude modulations of early potentials or both. The former would suggest that the parameters of the study rather than the dimension along which attention is varied accounts for the prior-entry effect. The latter would be more consistent with McDonald et al.’s (2005) findings, and suggest instead that the behavioural enhancements conferred by spatial attention and by attention to sensory modalities are brought about by different underlying modulatory mechanisms despite giving rise to similar behavioural effects.

## Methods

### Participants

Fifteen participants were recruited from the academic community of students and postdoctoral fellows at the University of Oxford. ERP data from one participant were very noisy and were, therefore, excluded. Analyses of the behavioural and electrophysiological data were carried out on the same group of 14 participants (10 males and 4 females, 13 right-handed, and 1 left-handed, ages ranging between 19 and 29 years). The participants received £20 remuneration for their participation in this study. All of the participants had normal tactile sensitivity and normal or corrected-to-normal vision by self-report. Each recording sessions lasted for about 2 h including electrode setup and breaks.

### Apparatus and materials

The experiment took place in a dark, electrically shielded, and sound-attenuated testing booth. Two tactile and two visual stimulators were triggered by Presentation 05 (Neurobehavioural Systems, Albany, California, version v 0.8) together with a custom-built interface box that was connected to the parallel port of the task-presentation computer. The visual and tactile stimuli were delivered to the dorsal medial phalynxes of the index fingers (or in close proximity). The visual and tactile stimuli both consisted of very brief 10-ms pulses as measured by a light sensor or a microphone. The visual stimuli consisted of the illumination of a red light-emitting diode (LED). Tactile stimulation consisted of taps by small plastic rods that were moved by means of small solenoids. The tactile stimulators (Heijo Research Electronics, London, UK) were suspended by adjustable rods against the participant’s fingertips, and weights were used to maintain a constant pressure against the skin surface. The participants’ hands were placed in a stable position within a specially made hand-shaped cast.

A permanently illuminated central fixation point, consisting of a red LED, was placed 42 cm directly in front of the participant. The participants’ hands, and associated LEDs and tactile stimulators, were placed one to either side of the fixation point, at a visual angle of 20° below fixation. To mask any sounds associated with the operation of the tactile stimulators, white noise (65 dB) was delivered centrally and earplugs (LaserLite^®^, San Diego California; noise reduction rate 32 dB) were worn. The participants were instructed to perform the experiment without moving their eyes. Furthermore, eye movements were monitored with an ISCAN^®^ ETL-400 eye tracker. Participants responded by lifting their feet off of the footpedals placed under their left and right foot.

### Design and procedure

Two peripheral stimuli were presented for 10 ms, separated by a variable stimulus onset asynchrony (SOA). The participants were instructed to determine whether the first stimulus was visual or tactile, and responded by lifting their toes off of the footpedal on the specified side (left pedal—touch, right pedal—vision, and vice versa for counterbalancing). Two types of trials were presented. Bilateral trials consisted of one stimulus being presented in each spatial location (left and right), and unilateral trials consisted of two stimuli being presented sequentially from the same spatial location (left or right).

The participants were introduced to the experiment by means of a written description and a brief practice session. They then completed 20 experimental blocks of trials. These were divided into two spatial-attention conditions, where attention was biased to stimuli presented in the left or right external hemispace. The order of presentation of the attention conditions was counterbalanced across participants. A short break was introduced between each block of trials within each condition, and a longer break was allowed between the two attention conditions to ensure maximal alertness on the part of the participants.

In the two conditions, attention was biased either toward the left or the right side of space by means of verbal instruction by the experimenter, and by including a higher frequency of stimuli at that location. In each condition, two-thirds of the trials were bilateral, while one-third were unilateral (i.e., containing only stimuli at the “attended” location; see Table 1 for details). Unilateral trials consisted of visual flashes and tactile taps separated by one of five SOAs centered on zero: 0, ±35, and ±150 ms in a similar manner to that reported in an earlier study (Vibell et al. 2007). Bilateral trials in the present study SOAs were centered on the PSSs, which had been established in a separate behavioural experiment in Vibell et al.’s (2007) study. The SOAs used were: −90 (tactile precedes visual), 25, 60, 95, and 210 ms. Unilateral and bilateral trials, with visual and tactile leading stimuli, at each SOA, were randomly intermixed, and appeared in an unpredictable order.

**Table 1:** Number of trials for the five different SOAs (negative SOAs indicate that the tactile stimulus was presented before the visual), for both unilateral and bilateral trials separated by attention condition

SOA (ms)	Attend left					Attend right				
	−90	25	60	95	210	−90	25	60	95	210
Bilateral VT and TV	50	100	100	100	50	50	100	100	100	50
Unilateral VT and TV	25	50	50	50	25	25	50	50	50	25

All values in milliseconds

There were ten blocks of trials per condition (attend left and attend right) with each block lasting for approximately 2–3 min. Each block contained 60 trials giving rise to a total of 600 trials in each attention condition. There were 400 bilateral trials, divided equally into left-first and right-first trials, but with a greater proportion of trials being presented at the middle three SOAs (100 trials each) as compared to the two SOAs furthest away from objective simultaneity (50 trials each). This translated into 50 vision-first trials and 50 touch-first at the 25-, 60-, and 95-ms SOAs; and 25 vision-first trials and 25 touch-first trials at the −90 and 210-ms SOAs. The 200 unilateral trials used a similarly-proportioned distribution of trials: 50 trials with simultaneously-delivered visual and tactile stimuli (0-ms SOA); 100 trials with equally distributed vision-first and touch-first stimuli at 35-ms SOA; and 50 trials with vision-first and touch-first stimuli at the 150-ms SOA.

Each trial started with the presentation of two stimuli, one in each sensory modality. Participants then responded according to the modality of the stimulus that they thought occurred first. They were told that the accuracy of their responses was more important than the speed, but that they should nevertheless respond as rapidly as they could. The next trial did not start until a response had been made and a random intertrial interval between 1500 and 2000 ms had elapsed.

The percentages of bilateral trials in which participants judged that the left/right stimulus was presented first at each SOA were computed, and subsequently normalized into Z-scores (see Spence et al. 2001). The PSS for each participant was calculated by fitting the best-fitting straight line through the Z-scores across the five SOAs (−90 to 210 ms), and interpolating the value of 50/50 responses (see Cohen et al. 1999). These values were analyzed using a two-way repeated-measures analysis of variance (ANOVA) with the factors of attention (attend and ignore) and side of stimulation (left and right) or in the bilateral conditions with the factors of attention (attend and ignore) and type of stimulation (unilateral and bilateral).

### ERP recordings

EEG was recorded with Ag–AgCl electrodes from 34 scalp electrodes (Easy Cap, Herrsching-Breitbrunn, Germany; NuAmps digital amplifiers, Neuroscan, El Paso, Texas). Additional electrodes served as ground (AF_Z_), reference, and electrooculogram (EOG) channels. During recordings, the right mastoid (A2) was used as the active reference. Subsequently, the data were re-referenced offline to the digital average of both mastoids [(A1 + A2)/2]. Electrode impedance was kept below 5 kΩ. The horizontal EOG (HEOG) was recorded from the outer canthi of both eyes, and the vertical EOG (VEOG) was recorded from below and above the right eye. All recordings were sampled with an A/D rate of 500 Hz and subsequently filtered with a 40-Hz low-pass filter (DC-40 Hz).

As in Vibell et al.’s (2007) study, the present experiment was designed to analyze ERPs elicited by visual stimuli only. Because of the smaller amplitudes of potentials associated with vibrotactile as compared to visual stimuli, and because of the risk of contamination of the tactile potentials by the potentials evoked by preceding visual stimuli at most SOAs, analysis of tactile ERPs was not carried out. Furthermore, only bilateral trials were of interest. Trials with unilateral stimulation were only included in the experimental design to manipulate spatial attention and hence were not included in the ERP analysis.

EEG and EOG analysis was performed offline using Scan 4.3 (Neuroscan, El Paso). The raw data were epoched into periods starting 200 ms prior to the onset of the visual stimulus and continuing until 822-ms post-stimulus onset. The ERPs were measured with the pre-stimulus interval as a baseline. Trials including eye blinks or large eye movements, measured as large voltage deflections on the HEOG or VEOG channels (±50 µV), were automatically removed. In addition, epochs containing potentials above ±150 µV in any channel were also removed to avoid large drifts in the signal. Epochs were also inspected visually to ensure that all eye movements, drift, or excessive alpha activity had been eliminated.^2^ After artifact rejection, ERPs for visual stimuli in each attention condition, side, and SOA consisted of an average of 33 trials for the three middle conditions and 17 trials for the two extreme SOA conditions. When stimulus side and SOA were collapsed (see below), the average number of trials was 266, ranging between 172 and 359 across participants.

To test for the effects of spatial attention on brain activity elicited by visual stimuli in this TOJ task, the potentials elicited by visual stimuli were analyzed at the electrodes and time periods, where they were most pronounced. This approach is the same as that followed by Vibell et al. (2007), and, therefore, facilitates comparisons across the two studies. To measure any modulations in the amplitude of brain activity, mean amplitudes were obtained around the average time for identifiable potentials to peak across participants. Mean amplitudes were measured over a narrow band around the average peak latency of the potential to minimize the possible contribution of brain activity related to other overlapping brain potentials. To measure the timing of brain activity over successive stages of information processing, the peak latencies for identifiable potentials were measured and compared. Peak latencies were identified by a simple automated computer algorithm, which defined the absolute maximum or minimum voltage value for positive or negative potentials within a temporal window, respectively. The temporal windows used to identify these peak latencies were enlarged relative to those used in mean-amplitude measures, to accommodate the variability in the timing of potential peaks across participants. The results were subsequently inspected visually to ensure that the automated algorithm was functioning properly and that the measurements were not contaminated by excessive noise or drift.

Mean amplitudes and peak latencies of the first identifiable visual potential P1 were analyzed at electrodes O1/2, PO3/4, and PO7/8 between 100 and 200 ms (latencies) and 140 and 160 ms (amplitudes). The later visual N1, P2, and N2 potentials were analyzed at electrodes O1/2, PO3/4, PO7/8, P3/4, and P7/8 in the following ranges for latencies: 150–250 ms (N1), 200–300 ms (P2), and 250–350 ms (N2) and in the following ranges for amplitudes: 180–220 ms (N1), 240–360 ms (P2), and 280–300 ms (N2). The late P3 potential was identified and analyzed at electrodes C3/Z/4, CP3/Z/4, and P3/Z/4; between 300 and 600 ms for latencies and between 350 and 450 ms for amplitudes. The effects of the attentional manipulation upon the mean amplitude and latency of the potentials were assessed using repeated-measures ANOVAs, testing for the factors of attention (left and right), stimulus side (left and right), SOA (−90, 25, 60, 95, and 210 ms), scalp hemisphere (contralateral, ipsilateral, and midline where relevant), and electrode location. To control for possible violations of sphericity, Greenhouse–Geisser adjustments were applied to the degrees of freedom where necessary.

**Fig. 1:**
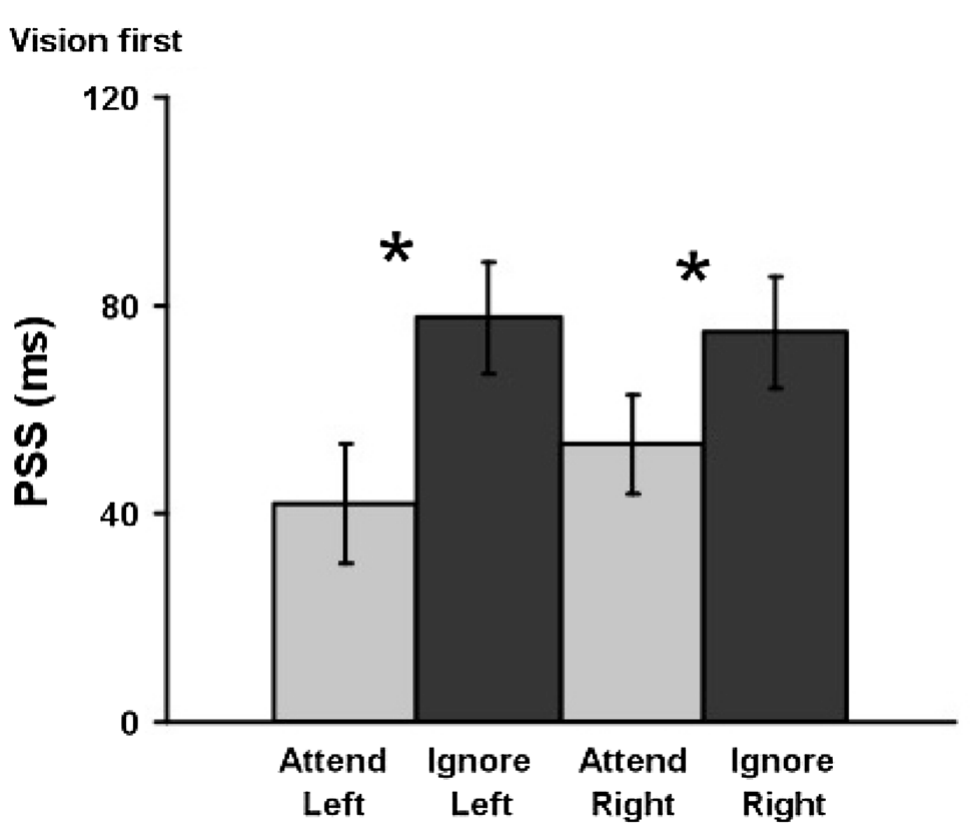
Prior-entry effect. Effects of spatial attention on the mean PSS, the amount of time by which the visual stimulus had to lead the tactile stimulus in order for the two to be perceived as simultaneous. *Asterisks* indicate a significant difference (*p* < 0.001, two-way *t* test) that was observed between the attention conditions. *Error bars* indicate the standard error of the means

Main effects and interactions including the attentional manipulation were the main interest. Latency shifts from attention were considered as evidence of prior entry. To center the SOAs on the PSS, vision was presented an average of 60 ms before touch. Therefore, we only looked at visual potentials as tactile potentials were too contaminated by the earlier presented visual stimulation. To rule out any influence of possible overlap from tactile potentials upon the latency measures of visual potentials at any given SOA, only effects that did not interact with the SOA factor were considered as indicators of prior entry. Attention effects that did not interact with the SOA factor were followed up by a simpler analysis, which maximized signal to noise. Since neither side nor SOA interacted with attention, ERPs elicited by the left and right stimuli at each of the five SOAs were combined using weighted averaging according to the number of trials in each condition. ERPs from left- and right-side stimulation were combined in a way that preserved their position relative to the stimulus location (contralateral versus ipsilateral). Electrodes were renamed as contralateral or ipsilateral to the stimuli for averaging (Fig. 1). To be consistent with Vibell et al. (2007), the data were visualized using the Cartool software by Denis Brunet (http://brainmapping.unige.ch/cartool).

## Results

**Fig. 2:**
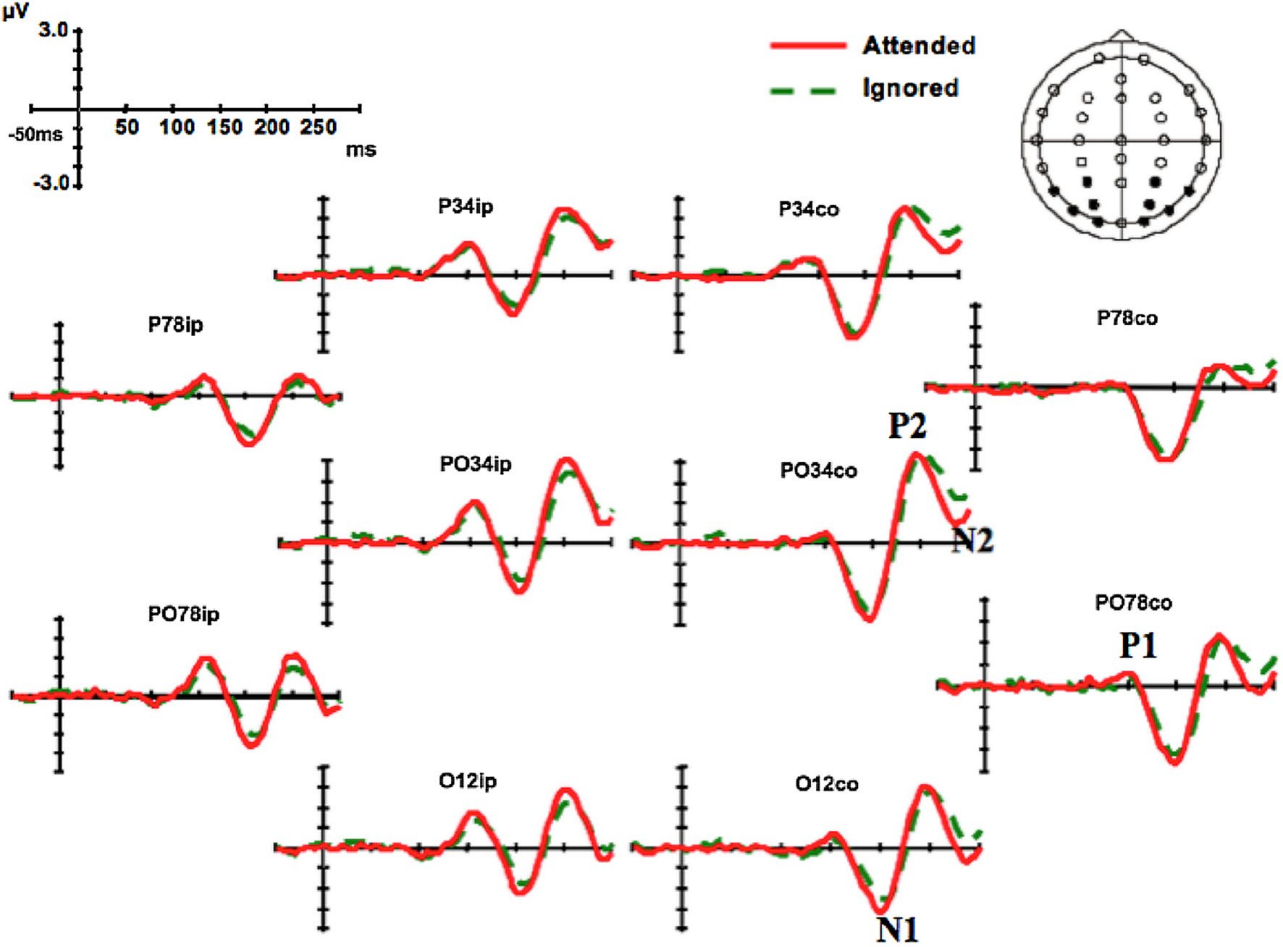
Early ERP amplitude and latency modulations. Significant amplitude effects were observed for the P1, while the N1, P2, and N2 showed significant attention effects for both amplitudes and latencies

A two-way repeated-measures ANOVA with attention and side as factors showed that attending to a location significantly shifted the PSS [*F*(1,13) = 28.2, *p* < 0.001, *η* ^2^ = 2.17]. For visual and tactile stimuli to be perceived as simultaneous, the visual stimuli would have had to precede the tactile stimuli by 48 ms when the location, where the visual stimulus was presented, had been attended, and by 76 ms when the location of the visual stimulus was ignored/unattended according to the mean PSSs (see Fig. 2). This constituted a shift in the PSS of 28 ms by attention. This prior-entry effect was confirmed by post hoc follow-up analysis using *t* tests [*t*(1,13) = −5.3, *p* < 0.001]. Neither a significant main effect of stimulus side nor any interaction was observed (all *F*s < 0.5). Although non-significant, differences for attended versus ignored stimuli on the left side (36 ms) were somewhat larger than the differences observed on the right side (21 ms), again in line with Spence et al.’s (2001, Experiments 3 and 4) previous psychophysical findings. The difference in just noticeable differences (JNDs) between unimodal and bimodal stimulus pairs when the location of the visual stimulus was attended as compared to when it was ignored was not significant, nor were there any effects for side or interactions (all *F*s < 0.5). JNDs of 65 ms when the location of the stimuli was attended and of 67 ms when it was ignored indicated similar difficulties in the detection of the stimuli.

A follow-up comparison was made between the present set of data and the results when the attention of participants was directed to a particular sensory modality (i.e., vision or touch; see Vibell et al. 2007). The two studies were compared using a mixed-effects ANOVA comparing the PSSs across the between-participants factor of Attended Dimension (modality versus space), and the within-participants factors of attention (attended and ignored), and side (left and right). The ANOVA revealed a significant main effect of attention [*F*(1,13) = 33.91, *p* < 0.001, *η* ^2^ = 2.61], but no interactions involving the attended dimension factor or any of the other factors [all *F*’s < 1]. These results demonstrate that there were no significant differences in the magnitude of the prior-entry effects reported psychophysically in the two studies (28 versus 38 ms) nor were there any effects of side of stimulation.

### ERP effects

**Fig. 3:**
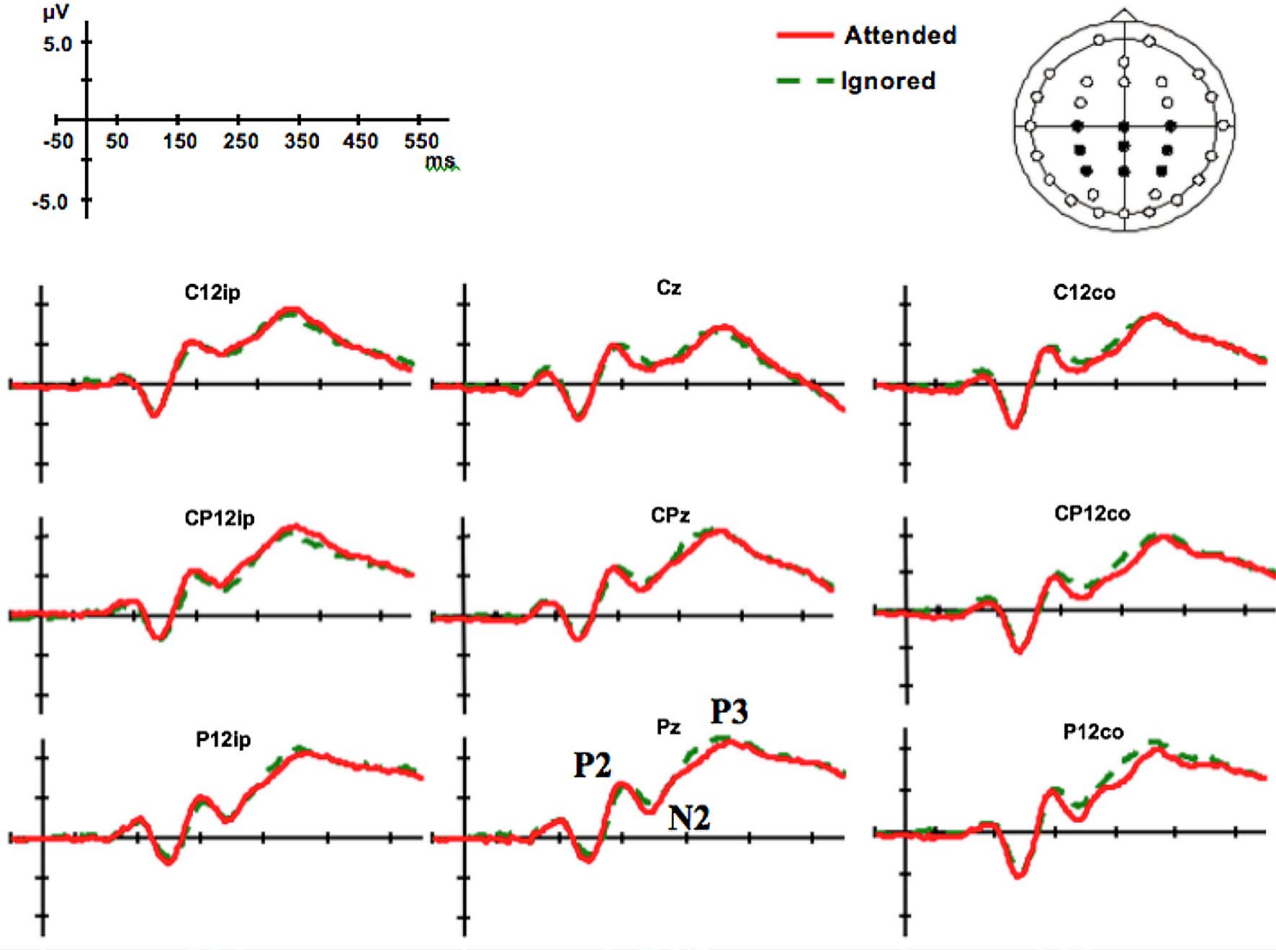
Late ERP amplitude and latency modulations. Waveforms for the attended stimuli (*solid lines*) showed significant differences from the waveforms for ignored stimuli (*dashed lines*) for amplitudes at P2, N2, and P3 and for latencies at P2 and P3

The ERP data from one participant contained an excessive degree of noise and were excluded from analysis (a total of 43 trials as compared to between 172 and 359 for the rest of the participants). For the remaining participants, visual stimuli within bimodal trials elicited small visual P1, and clear N1, P2, N2, and late P3 potentials. Visual potentials had a characteristic lateral posterior distribution, and because of the dim and very peripheral nature of the stimulation used (e.g. Störmer et al. 2009; Tünnermann and Scharlau 2016; Akyürek and de Jong 2017), occurred relatively late in this task (see Fig. 3). The P1 potential was largest over PO3/4, PO7/8, and O1/2 electrodes and showed an unusual ipsilateral predominance. The N1, P2, and N2 had a slightly broader lateral posterior distribution, over electrodes O1/2, PO3/4, PO7/8, P3/4, and P7/8. The P3 potential had a broad distribution, which was maximal over the central–parietal region of the scalp over electrodes C3/Z/4, CP3/Z/4, and P3/Z/4.

#### Peak latencies

Shifts in peak latencies were observed for late potentials (P2, N2, and P3), but not for the early P1 and N1 potentials. The peak latency of the P1 potential failed to show any effects of stimulus side or SOA, nor did any of these factors interact with attention (all *p*’s > 0.11). Accordingly, the stimulus side and SOA factors were collapsed, and a simpler P1 analysis confirmed the lack of attentional effects on the latency of the P1 potential.

We did not see attentional effects for the N1 potential including between attention, electrode, and hemisphere [*F*(4,52) = 2.51, *p* = 0.053, *η* ^2^ = 0.19]. A main effect for SOA revealed differences between N1 peak latencies across SOA conditions, with the latencies being generally longer in the 60-ms SOA condition and becoming progressively shorter toward the extreme SOA values [*F*(2.55,33.12) = 10.40, *p* < 0.001, *η* ^2^ = 0.80]. A significant three-way interaction between SOA, stimulus side, and hemisphere [*F*(4,52) = 4.38, *p* < 0.05, *η* ^2^ = 0.34] further qualified these effects by demonstrating earlier peaks for the earliest SOA contralaterally when the stimuli were presented on the left side. Since we did not observe interactions between attention and SOA, the stimuli were collapsed over SOA and we performed a post hoc analysis. The results again failed to show a significant interaction between attention, electrode, and hemisphere. The interaction between attention and stimulus side also failed to show stronger attentional effects for the right stimulus side [*F*(1,13) = 3.32, *p* = 0.092, *η* ^2^ = 0.26].

Attentional effects on latency became reliable for the P2 potential, where a main effect of attention was observed [*F*(1,13) = 6.43, *p* < 0.05, *η* ^2^ = 0.49]. Attending to the relevant spatial location resulted in a shift of the P2 peak 6 ms earlier from 259 to 253 ms. The latency shifts were qualified by a complex four-way interaction between attention, stimulus side, electrode, and hemisphere suggesting that latencies are significantly earlier for attended stimuli from the left side over the right occipital hemisphere [*F*(4,52) = 3.55, *p* < 0.05, *η* ^2^ = 0.27]. However, no interaction occurred between the factors of attention and SOA, thus suggesting that the change in P2 latency could not be attributed to a differential temporal overlap between components. A post hoc follow-up analysis similar to Vibell et al. (2007) with the data collapsed over SOA confirmed the main effect of attention, as well as the four-way interaction between attention, stimulus side, electrode, and hemisphere.

Significant latency shifts attributable to attention were also observed in the N2 potentials, but these interacted with the SOA factor and, therefore, could not be unambiguously attributed to prior entry. An interaction between attention, SOA, and electrode [*F*(16,208) = 1.73, *p* < 0.05, *η* ^2^ = 0.13] revealed that latency shifts were particularly pronounced over P3/4 and P7/8 electrodes for the 60- and 95-ms SOAs (3–11 ms). An additional significant interaction between attention, stimulus side, and electrode showed that the strongest latency shifts occurred when the stimuli were presented on the right side [*F*(4,52) = 3.36, *p* < 0.05, *η* ^2^ = 0.26]. There was also a four-way interaction between SOA, attention, stimulus side, and electrode showing that attentional effects were strongest for the P3/4 and P7/8 electrodes for the 50- and 85-ms SOAs when stimuli were presented on the right side [*F*(16,208) = 1.96, *p* < 0.05, *η* ^2^ = 0.15].

The P3 potential showed longer latencies for attended stimuli than for ignored stimuli over midline and right-sided electrodes (12–17 ms; see Fig. 3), as revealed by the interaction between attention and hemisphere [*F*(2,26) = 9.44, *p* = 0.001, *η* ^2^ = 0.73]. The midline attentional effects were supported by a four-way interaction between attention, SOA, hemisphere, and electrode, with these effects being more pronounced over the central electrodes at the −90-ms SOA. The interaction over central electrodes at −90 ms, on those trials, where touch was presented before vision, indicated that the attentional effects might also interact with the tactile stimulation. A statistical trend toward an interaction between attention, stimulus side, electrode, and hemisphere revealed a complex pattern of P3 latency modulation, with ignored stimuli eliciting a slightly earlier P3, particularly over the contralateral central and centro-parietal regions [*F*(4,52) = 2.48, *p* = 0.06, *η* ^2^ = 0.19].

A Pearson’s correlation analysis tested whether there was a linear relationship between the early attentional differences in the peak latencies and the PSS values obtained behaviourally. No correlations were observed between the differences in visual peak latencies and the differences in PSSs (all *r* < 0.389).

#### Mean amplitudes

The mean amplitude of the P1 potential was modulated by attention, but in a way that interacted with the SOA. A significant interaction between attention and SOA [*F*(4,52) = 2.95, *p* < 0.05, *η* ^2^ = 0.23] indicated that the modulations were particularly strong at the more extreme SOAs. No interactions between SOA, attention, and stimulus side [*F*(2.19,28.42) = 2.41, *p* = 0.061, *η* ^2^ = 0.19]; and between SOA, attention stimulus side, and electrode [*F*(2.91,37.82) = 4.77, *p* = 0.055, *η* ^2^ = 0.37], were observed when the stimuli came from the left and especially at the PO7/8 electrodes. Interactions involving SOA revealed some differences between the intervals with strong activations in the −90-ms SOA for all electrodes except the O1/2 and PO7/8 electrodes as indicated by a significant interaction between SOA and electrode [*F*(2.40,31.13) = 3.24, *p* < 0.05, *η* ^2^ = 0.25]. Different effects between SOAs were further qualified by significant interactions between SOA, stimulus side, and hemisphere [*F*(4,52) = 2.95, *p* < 0.05, *η* ^2^ = 0.23] and SOA, electrode, and hemisphere [*F*(5.04,65.46) = 5.17, *p* < 0.001, *η* ^2^ = 0.40]. This revealed particularly strong activations for the 25- and 210-ms SOAs when the stimuli were presented from the left over the PO4 and PO8 electrodes.

Attention effects were also observed for the N1 potential. A significant interaction between attention and electrode, indicated that these were differentially distributed over the lateral posterior electrodes, being more pronounced over PO7/8 and P7/8 [*F*(1.56,20.22) = 2.42, *p* < 0.05, *η* ^2^ = 0.19]. The N1 amplitude modulation by attention also interacted with SOA. The N1 showed attentional interactions between SOA, attention, and stimulus side [*F*(3.26,42.35) = 4.52, *p* < 0.05, *η* ^2^ = 0.35], as well as a four-way interaction between SOA, attention, stimulus side, and electrode [*F*(3.51,45.68) = 2.74, *p* < 0.05, *η* ^2^ = 0.21]. Here, the three middle SOAs (25, 60, and 95 ms) revealed stronger attentional effects particularly for those stimuli presented on the right side. A main effect of SOA on the mean amplitude of the N1 was also documented [*F*(2.14,27.85) = 7.96, *p* < 0.001, *η* ^2^ = 0.61], with the N1 in the −90-ms SOA condition differing most from those at the other SOAs. This was further supported by interactions between SOA and electrode [*F*(3.93,51.14) = 7.02, *p* < 0.05, *η* ^2^ = 0.54]; SOA, stimulus side, and electrode [*F*(4.58,59.51) = 1.89, *p* < 0.05, *η* ^2^ = 0.15]; SOA, stimulus side, and hemisphere [*F*(2.59,33.73) = 2.86, *p* < 0.05, *η* ^2^ = 0.22]; SOA, electrode, and hemisphere [*F*(5.14,66.75) = 5.48, *p* < 0.001, *η* ^2^ = 0.42]; and SOA, stimulus side, electrode, and hemisphere [*F*(3.72,48.34) = 1.91, *p* < 0.05, *η* ^2^ = 0.15]. The effects of SOA were particularly pronounced for stimuli presented on the left over the PO8 electrode.

Just as for the earlier potentials, the mean amplitude of the P2 was enhanced by attention. This enhancement was particularly pronounced over the left hemisphere [*F*(1,13) = 4.84, *p* < 0.05, *η* ^2^ = 0.37] and was expressed in all electrode pairs except the P7/8 [*F*(1.9,24.1) = 3.82, *p* < 0.05, *η* ^2^ = 0.30]. In addition, a three-way interaction between attention, electrode, and hemisphere [*F*(1.9,24.0) = 2.26, *p* < 0.05, *η* ^2^ = 0.18] was observed, showing that these attentional effects were more pronounced over the right hemisphere. However, the amplitude modulations also interacted with the SOA condition in complex ways. There was a trend toward an interaction between SOA, attention, and stimulus side [*F*(2.5,32.0) = 2.29, *p* = 0.072, *η* ^2^ = 0.18], and a significant interaction between SOA, attention, stimulus side, and electrode [*F*(1,13) = 4.87, *p* < 0.05, *η* ^2^ = 0.37]. This indicated that stimuli from the left in the 210-ms SOA over the PO3/4 electrodes elicited particularly strong amplitudes. In addition, a main effect for SOA indicated that the P2 potentials were larger over the extreme SOAs [*F*(1.9,25.0) = 3.91, *p* < 0.05, *η* ^2^ = 0.30]. The effect of SOA interacted with several variables other than attention in a complex manner. Interactions were observed between SOA and hemisphere [*F*(2.61,33.90) = 3.40, *p* < 0.05, *η* ^2^ = 0.26]; SOA and electrode [*F*(4.60,59.81) = 6.23, *p* < 0.001, *η* ^2^ = 0.48]; SOA, stimulus side, and electrode [*F*(4.82,62.61) = 2.00, *p* < 0.05, *η* ^2^ = 0.15]; and SOA, electrode, and hemisphere [*F*(5.31,69.02) = 2.65, *p* = 0.001, *η* ^2^ = 0.20]. Overall, the P2 was larger for the 95-ms SOA, particularly over the PO4 electrode.

Attention once again enhanced the amplitude of the N2 potential over selected electrodes, but the effects further interacted with the SOA condition. An interaction between attention stimulus side, electrode, and hemisphere [*F*(2.4,30.9) = 2.58, *p* < 0.05, *η* ^2^ = 0.20] revealed enhancements of the N2 by attention, particularly for right visual-field stimuli, over the PO3/4 and P3/4 electrodes. The influence of SOA upon the attention effects was highlighted by significant interactions between SOA, attention, and stimulus side [*F*(4,52) = 5.85, *p* = 0.001, *η* ^2^ = 0.45]; and SOA, attention, stimulus side, and electrode [*F*(4.6,60.0) = 2.58, *p* = 0.001, *η* ^2^ = 0.20]. Attentional effects on N2 were largest for right-sided stimuli at the 25-ms SOA, particularly over the P3/4 and PO3/4 electrodes. Effects of SOA that did not interact with attention were also obtained. There was, for instance, a significant main effect of SOA [*F*(2.1,27.4) = 11.33, *p* < 0.001, *η* ^2^ = 0.87]; as well as interactions between SOA and electrode [*F*(2.2,28.5) = 4.28, *p* < 0.05, *η* ^2^ = 0.33]; SOA, stimulus side, and hemisphere [*F*(2.2,28.5) = 4.28, *p* < 0.05, *η* ^2^ = 0.33]; SOA, electrode, and hemisphere [*F*(2.2,28.5) = 4.28, *p* < 0.05, *η* ^2^ = 0.33]; and SOA, stimulus side, electrode, and hemisphere [*F*(2.2,28.5) = 4.28, *p* < 0.05, *η* ^2^ = 0.33]. Overall, the N2 potential was largest over the PO3/4 and P3/4 for all SOAs except the −90 SOA, particularly in the left hemisphere when stimuli were presented on the right side.

Analysis of the P3 revealed a significant main effect for attention [*F*(1,13) = 5.63, *p* < 0.05, *η* ^2^ = 0.43]. Attended stimuli elicited a significantly smaller P3 overall (see Fig. 3). However, the pattern of P3 modulation differed over the different regions of the scalp. Interactions between attention and hemisphere [*F*(1.2,15.7) = 3.51, *p* < 0.05, *η* ^2^ = 0.27]; and between attention and electrode [*F*(1.0,13.5) = 5.87, *p* < 0.05, *η* ^2^ = 0.43], indicated that the attenuation of the P3 occurred mainly over midline and right-hemisphere electrodes over parietal regions. SOA again influenced these attention-related effects. Interactions occurred between SOA, attention, and stimulus side [*F*(2.9,37.7) = 3.20, *p* < 0.05, *η* ^2^ = 0.25]; SOA, attention, and electrode [*F*(3.3,42.7) = 4.08, *p* < 0.001, *η* ^2^ = 0.32]; SOA, attention, stimulus side, and electrode [*F*(2.1,26.9) = 2.43, *p* < 0.05, *η* ^2^ = 0.19]; and SOA, attention, and hemisphere [*F*(4.1,53.7) = 2.14, *p* < 0.05, *η* ^2^ = 0.16]. These interactions showed stronger attentional effects for the −90-, 25-, and 60-ms SOAs, particularly for midline and parietal electrodes. Effects of SOA that did not interact with attention were also observed. There was a main effect of SOA [*F*(2.2,28.5) = 4.28, *p* < 0.05, *η* ^2^ = 0.33]; as well as interactions between SOA and hemisphere [*F*(3.9,50.4) = 13.80, *p* < 0.001, *η* ^2^ = 1.07]; SOA and electrode [*F*(1.3,23.7) = 9.36, *p* < 0.001, *η* ^2^ = 0.51]; SOA, stimulus side, and electrode [*F*(2.9,38.4) = 2.39, *p* < 0.05, *η* ^2^ = 0.18]; and SOA, electrode, and hemisphere [*F*(5.4,70.1) = 4.64, *p* < 0.001, *η* ^2^ = 0.36]. This set of interactions highlighted that the P3 potentials were most pronounced for the −90-, 25-, and 60-ms SOAs for midline- and left-hemisphere parietal electrodes.

## Discussion

The present study was designed to evaluate whether the particular dimension in which endogenous attention was oriented was responsible for the latency shifts in early perceptual processing observed by Vibell et al. (2007). The experiment reported here was identical to our previous study, except for the fact that the participants here had to focus their attention endogenously on a particular spatial location and decide on the modality of the stimulus that occurred first (to maintain orthogonality of the experimental design; cf. Spence et al. 2001). The results provide support for the claim that the dimension along which attention is oriented plays an important role in determining the neural mechanisms underlying any behavioural facilitation effects that are observed.

Behaviourally, the results of the present study were similar to those reported by Vibell et al. (2007), even though space and modality were transposed. Visual and tactile stimuli occurring at the attended spatial location were reported as occurring significantly earlier in time than those appearing at the ignored spatial location. The behavioural data from the present study, therefore, confirm the results from the paper by Vibell et al., as well as those from other earlier studies (see Spence and Parise 2010, for a review). The behavioural data were in line with earlier work (Spence et al. 2001, Experiments 3 and 4) in showing that spatial attention shifted the PSS toward the attended modality. This study used a simple PSS-based method for comparability with Vibell et al. (2007), but see also Alcalá-Quintana and García-Pérez (2013), García-Pérez and Alcalá-Quintana (2015), Krüger et al. (2016) for more detailed analysis approaches. (We will publish the data for further assessment by the modeling community.)

The pattern of behavioural prior-entry effects was similar to that observed in previous endogenous cuing studies. A behavioural prior-entry effect of 28 ms was observed here as compared to a shift of 38 ms in the study by Vibell et al. (2007). A between-studies comparison of the shift in PSS values, however, revealed that these effects were not significantly different. Despite showing a smaller prior-entry effect for spatial attention than in Spence et al. (2001) previous study (121 ms), the slightly smaller (though non-significant) difference between the present study and the study by Vibell et al. concurs with Spence and colleagues’ findings in suggesting a smaller effect in the spatial dimension than in the modality dimension. Further studies are needed, however, to confirm this.

The ERP results show both similarities and differences to those observed by Vibell et al. (2007). Spatial attention, within the context of the TOJ task, influenced the latency of potentials elicited by visual stimuli. The effects started later than those reported for modality-based attention by Vibell et al. with the P2 potential showing significantly earlier latencies in the attended as compared to the ignored stimulus conditions. The latency shifts occurred independently of the SOA, thus providing evidence in support of the occurrence of post-perceptual latency effects, and arguing against the effects being caused by artifacts or by an interaction between the visual and tactile evoked potentials. Latency modulations continued to be observed for even later potentials, but in this case, the effects of spatial attention interacted with the SOA conditions, making it difficult to rule out contamination of potential overlap from the successive stimuli as a possible explanation.

The ERP results from the present endogenous spatial-attention TOJ study highlight both similarities and differences with the findings reported by McDonald et al. (2005) using exogenous spatial attention directed to audio-visual TOJs. In their study, spatial attention did not significantly shift the latency of the earlier perceptual potentials (P1 and N1) as in Vibell et al. (2007). Instead, they found latency modulations only from post-perceptual stages of information processing (P2 and onwards). The present study showed similar shifts to McDonald et al. occurring in the P2 potential. We found a 6-ms shift for the P2 potential, which is very similar to the 5-ms difference observed by McDonald and colleagues. We did not, however, see the enhanced contralateral positivity reported by McDonald et al. (2005; also Störmer et al. 2009; and McDonald et al. 2013).

By comparing the results in the present experiment to those reported by Vibell et al. (2007), it can be concluded that the dimension along which attention is oriented can influence the ability to detect shifts in the latency of early visual potentials. However, additional experimental factors, other than the dimension of attention (e.g. response bias), may also influence the ability to observe perceptual prior-entry effects in TOJ experiments. Vibell et al.’s (2007) study refutes such claims with quite early attentional modulations (P1), which are unlikely to have stemmed from response bias which is thought to occur at later cognitive processing stages. This leaves open, however, the question of whether later components are influenced by response bias.

The P3 (not analyzed in McDonald et al.’s 2005, study) exhibited a reverse latency effect. This has been observed in other studies using spatial attention, though it is nevertheless still somewhat unusual (Eimer 1994; Griffin et al. 2002). One of the interactions suggested that attentional effects were the strongest when touch is presented 90 ms before vision and particularly over somatosensory areas, suggesting that the tactile stimuli might interact with the effect of the later potentials. Since touch is perceived on average 60 ms earlier than vision, it is likely that the tactile stimulation can interfere with the later visual potentials causing the interactions by SOA that we observed. In addition, several studies have demonstrated that the amplitude of the P3 depends on target probability (e.g., Duncan-Johnson and Donchin 1977; Kutas et al. 1977). The P3 increases as a function of decreasing the probability of occurrence of the relevant stimulus. Therefore, it is perhaps not so surprising that in the present study, using an increased number of stimuli in the attended location induced a decreased P3, canceling out any potential attention effects. Using the same type of paradigm, the study by Vibell et al. (2007) showed P3 amplitudes that just failed to reach significance for attention. In that study, the effects were stronger for the P3 when stimuli were attended, but might have been even stronger with another attentional manipulation, where attention was not directed based on increasing the frequency of stimuli.

The waveforms observed in the present study were very similar to those observed by Vibell et al. (2007), where attention was oriented to a specific sensory modality. However, compared to their effects of modality-based attention during the performance of a TOJ task, more amplitude modulation of the visual potentials was observed in the present study. These effects consistently interacted with the SOA conditions, thus making their interpretation in this context somewhat problematic. Nevertheless, their occurrence, combined with the later-emerging effects on the peak latency of visual potentials, clearly shows that the mechanisms of neural modulation by spatial attention differ from those by modality-based attention (see also Störmer et al. 2009; Grabot and van Wassenhove 2017).

Research comparing dimensions of attention (e.g., attention to spatial locations or sensory modalities) have shown that slightly different mechanisms can underlie similar behavioural effects, as in the case of attention to spatial locations and temporal intervals (see Nobre and Silvert 2008). Despite using similar paradigms, mostly based on Posner and Rothbart (1980) influential attentional orienting task, these studies showed different stages of attentional modulation and even latency changes in later potentials for the case of temporal attention (Griffin et al. 2002). The Posnerian cuing paradigm typically uses spatial-attentional manipulations, which may explain why early amplitude enhancements have been observed in many of previous studies. The latency shift in the studies investigating attention to time (Correa et al. 2006; Griffin et al. 2002; see also Miniussi et al. 1999) was observed for the P3 possibly reflecting the cognitive nature of their temporal manipulation. Correa and colleagues also found a speeding-up (by 9 ms) in the N2 potential by temporal attention. They attributed the earlier N2 shift to a higher demand on perceptual processing, which is also the case for TOJ studies. This is comparable to the results here, showing perceptual latency shifts, probably due to the perceptual nature of the discrimination. The nature of the task may, therefore, interact with the attentional dimension in the modulation of ERPs.

The results of the research reported here suggest that attention directed to spatial locations operates slightly later in time than does attention to sensory modalities (see also Spence et al. 2001). Though, it should be borne in mind that enhanced contralateral positivities reported in previous studies of spatial attention consistently emerged in the interval of the P1 (e.g., Nobre and Silvert 2008). In summary, despite using the same task and parameters as Vibell et al. (2007), and only switching the dimension of attention and the dimension of response, latency modulations were observed at slightly later stages. This suggests that attention to spatial locations modulates peak latencies slightly later than attention to sensory modalities. Therefore, the present data suggest that the type of attention induced the exceptionally early latency shifts in Vibell et al.’s (2007) study. The findings show the ability of ERPs to discriminate different neural mechanisms underlying what looks like the same behavioural effect. Future work should refine our understanding of how attention to spatial locations and to sensory modalities influences early sensory processing differently. It would be particularly interesting, for instance, to include a different attentional dimension for space (e.g., up/down) to be able to compare attention to spatial locations and to sensory modalities using the same response dimension (respond left/right) for both conditions.

## Footnotes

1 These include increasing latency shifts between cortical potentials and the various stages from perception to response. McDonald et al. (2012, p. 516) suggested that the results of “a single participant with an implausibly large latency difference could have biased results”. However, statistical analysis would not allow a single participant to create a significant result. This conclusion was verified with a second analysis using Cartool (Michel Lab, University of Geneva, Switzerland). Their last concern regarded overlap with tactile potentials: Since, vision was presented an average of 60 ms before touch to account for different rates of perception between the sensory modalities, it is unlikely that tactile potentials would influence the early visual potentials. Conversely, visual potentials presented 60 ms earlier would most certainly influence tactile potentials and were therefore not analyzed.

2 Because of the subjective nature of TOJs, it was not possible to remove trials according to behavioral errors.

## References

[CR1] Akyürek EG, de Jong R (2017). Distortions of temporal integration and perceived order caused by the interplay between stimulus contrast and duration. Conscious Cogn.

[CR2] Alcalá-Quintana R, García-Pérez MA (2013). Fitting model-based psychometric functions to simultaneity and temporal-order judgment data: MATLAB and R routines. Behav Res Methods.

[CR3] Anllo-Vento L, Hillyard SA (1996). Selective attention to the color and direction of moving stimuli: electrophysiological correlates of hierarchical feature selection. Percept Psychophys.

[CR4] Anllo-Vento L, Schoenfeld MA, Hillyard SA, Posner M (2004). Cortical mechanisms of visual attention: electrophysiological and neuroimaging studies. Cognitive neuroscience of attention.

[CR5] Boenke LT, Alais D, Ohl FW (2012) Visual N1 peak latency predicts individual location of point of subjective simultaneity and prior-experience in audiovisual temporal order judgments. Frontiers in Human Neuroscience. Poster at ACNS-2012. http://www.frontiersin.org/Journal/FullText.aspx?s=537&name=human_neuroscience&ART_DOI=10.3389/conf.fnhum.2012.208.00020

[CR6] Cohen S, Ward LM, Enns JT (1999). Sensation and perception.

[CR7] Correa A, Lupianez J, Madrid E, Tudela P (2006). Temporal attention enhances early visual processing: a review and new evidence from event-related potentials. Brain Res.

[CR8] de Ruiter MB, Kok A, van der Schoot M (1998). Effects of inter- and intramodal selective attention to non-spatial visual stimuli: an event-related potential analysis. Biol Psychol.

[CR9] Duncan-Johnson CC, Donchin E (1977). On quantifying surprise: the variation of event-related potentials with subjective probability. Psychophysiology.

[CR10] Eimer M (1994). “Sensory gating” as a mechanism for visuospatial orienting: electrophysiological evidence from trial-by-trial cuing experiments. Percept Psychophys.

[CR11] Eimer M (1996). ERP modulations indicate the selective processing as a result of transient and sustained spatial attention. Psychophysiology.

[CR12] Eimer M (1997). An event-related potential (ERP) study of transient and sustained visual attention to color and form. Biol Psychol.

[CR13] Eimer M, Forster B (2003). Modulations of early somatosensory ERP components by transient and sustained spatial attention. Exp Brain Res.

[CR14] Eimer M, Schröger E (1998). ERP effects of intermodal attention and cross-modal links in spatial attention. Psychophysiology.

[CR15] García-Pérez MA, Alcalá-Quintana R (2015). The left visual field attentional advantage: no evidence of different speeds of processing across visual hemifields. Conscious Cogn.

[CR16] Grabot L, van Wassenhove V (2017). Time order as psychological bias. Psychol Sci.

[CR17] Griffin IC, Miniussi C, Nobre AC (2002). Multiple mechanisms of selective attention: differential modulation of stimulus processing by attention to space or time. Neuropsychologia.

[CR18] Hillyard SA, Munte TF (1984). Selective attention to color and location: an analysis with event-related brain potentials. Percept Psychophys.

[CR19] Hillyard SA, Vogel EK, Luck SJ (1998). Sensory gain control (amplification) as a mechanism of selective attention: electrophysiological and neuroimaging evidence. Philos Trans R Soc Lond B Biol Sci.

[CR20] Hopf JM, Boelmans K, Schoenfeld MA, Luck SJ, Heinze HJ (2004). Attention to features precedes attention to locations in visual search: evidence from electromagnetic brain responses in humans. J Neurosci.

[CR21] Hotting K, Rosler F, Röder B (2003). Crossmodal and intermodal attention modulate event-related brain potentials to tactile and auditory stimuli. Exp Brain Res.

[CR22] Keetels M, Vroomen J, Murray MM, Wallace MT (2012). Perception of synchrony between the senses. The neural bases of multisensory processes.

[CR23] Krüger A, Tünnermann J, Scharlau I (2016). Fast and conspicuous? Quantifying salience with the theory of visual attention. Adv Cogn Psychol.

[CR24] Kutas M, McCarthy G, Donchin E (1977). Augmenting mental chronometry: the P300 as a measure of stimulus evaluation time. Science.

[CR25] Lester BD, Hecht LN, Vecera SP (2009). Visual prior entry for foreground figures. Psychon Bull Rev.

[CR26] Mangun GR, Hillyard SA (1991). Modulations of sensory-evoked brain potentials indicate changes in perceptual processing during visual-spatial priming. J Exp Psychol Hum Percept Perform.

[CR27] Matthews N, Welch L, Achtman R, Fenton R, FitzGerald B (2016). Simultaneity and temporal order judgments exhibit distinct reaction times and training effects. PLoS One.

[CR28] McDonald JJ, Teder-Salejarvi WA, Di Russo F, Hillyard SA (2005). Neural basis of auditory-induced shifts in visual time-order perception. Nat Neurosci.

[CR29] McDonald JJ, Green JJ, Störmer VS, Hillyard SA, Murray MM, Wallace MT (2012). Cross-modal spatial cueing of attention influences visual perception. The neural bases of multisensory processes.

[CR30] McDonald JJ, Whitman JC, Störmer VS, Hillyard SA (2013) Involuntary cross-modal spatial attention influences visual perception. In: Mangun GR (ed) Cognitive electrophysiology of attention: signals of the mind. Academic, Oxford, pp 82–94. doi:10.1016/B978-0-12-398451-7.00007-5

[CR31] Miniussi C, Wilding EL, Coull JT, Nobre AC (1999). Orienting attention in time: modulation of brain potentials. Brain.

[CR32] Miyazaki M, Kadota H, Matsuzaki KS, Takeuchi S, Sekiguchi H, Aoyama T, Kochiyama T (2016) Dissociating the neural correlates of tactile temporal order and simultaneity judgements. Scientific Reports 6(1). doi:10.1038/srep23323 10.1038/srep23323 PMC4827393 27064734

[CR33] Näätänen R (1986). The neural-specificity theory of visual selective attention evaluated: a commentary on Harter and Aine. Biol Psychol.

[CR34] Nobre AC, Silvert L, Marien P, Abutalebi J (2008). Measuring human cognition on-line with electrophysiological methods: the case of selective attention. Neuropsychological research.

[CR35] Olivers CNL, Hilkenmeier F, Scharlau I (2011). Prior entry explains order reversals in the attentional blink. Atten Percept Psychophys.

[CR36] Posner MI, Rothbart MK (1980). The development of attentional mechanisms. Nebr Symp Motiv.

[CR38] Santangelo V, Spence C (2008). Crossmodal attentional capture in an unspeeded simultaneity judgement task. Vis Cogn.

[CR39] Schneider KA, Bavelier D (2003). Components of visual prior entry. Cogn Psychol.

[CR37] Spence C, Driver J (1997). On measuring selective attention to an expected sensory modality. Percept Psychophys.

[CR40] Spence C, Parise C (2010). Prior entry: a review. Conscious Cogn.

[CR41] Spence C, Shore DI, Klein RM (2001). Multisensory prior entry. J Exp Psychol Gen.

[CR42] Spence C, McDonald J, Driver J, Spence C, Driver J (2004). Exogenous spatial-cuing studies of human crossmodal attention and multisensory integration. Crossmodal space and crossmodal attention.

[CR43] Störmer VS, McDonald JJ, Hillyard SA (2009). Cross-modal cueing of attention alters appearance and early cortical processing of visual stimuli. Proc Natl Acad Sci.

[CR1000] Talsma D, Kok A (2001). Nonspatial intermodal selective attention is mediated by sensory brain areas: evidence from event-related potentials. Psychophysiology.

[CR44] Talsma D, Kok A (2002). Intermodal spatial attention differs between vision and audition: an event-related potential analysis. Psychophysiology.

[CR45] Teder-Sälejärvi WA, Munte TF, Sperlich F, Hillyard SA (1999). Intra-modal and cross-modal spatial attention to auditory and visual stimuli. An event-related brain potential study. Cogn Brain Res.

[CR46] Titchener EB (1908). Lectures on the elementary psychology of feeling and attention.

[CR47] Tünnermann J, Scharlau I (2016). Peripheral visual cues: their fate in processing and effects on attention and temporal-order perception. Front Psychol.

[CR48] Tünnermann J, Petersen A, Scharlau I (2015). Does attention speed up processing? Decreases and increases of processing rates in visual prior entry. J Vis.

[CR49] Valdes-Sosa M, Cobo A, Pinilla T (1998). Transparent motion and object-based attention. Cognition.

[CR50] Vibell JF, Klinge C, Zampini M, Spence C, Nobre AC (2007). Temporal order is coded temporally in the brain: early ERP latency shifts underlying prior entry in a crossmodal temporal order judgment task. J Cogn Neurosci.

[CR51] Watt JD (1991). Effects of boredom proneness on time perception. Psychol Rep.

[CR52] Weiss K, Scharlau I (2009). Strategic influences on visual prior entry. Perception.

[CR53] Weiß K, Scharlau I (2011). Simultaneity and temporal order perception: different sides of the same coin? Evidence from a visual prior entry study. Q J Exp Psychol.

[CR54] Weiß K, Scharlau I (2012). At the mercy of prior entry: prior entry induced by invisible primes is not susceptible to current intentions. Acta Physiol (Oxf).

[CR55] West GL, Anderson AA, Pratt J (2009). Motivationally significant stimuli show visual prior entry: evidence for attentional capture. J Exp Psychol Hum Percept Perform.

[CR56] Woods DL, Alho K, Algazi A (1992). Intermodal selective attention. I. Effects on event-related potentials to lateralized auditory and visual stimuli. Electroencephalogr Clin Neurophysiol.

[CR57] Woods DL, Alho K, Algazi A (1993). Intermodal selective attention: evidence for processing in tonotopic auditory fields. Psychophysiology.

[CR58] Yates MJ, Nicholls MER (2009). Somatosensory prior entry. Percept Psychophys.

[CR59] Yates MJ, Nicholls MER (2011). Somatosensory prior entry assessed with temporal order judgments and simultaneity judgments. Attent Percept Psychophys.

[CR60] Zampini M, Shore DI, Spence C (2005). Audiovisual prior entry. Neurosci Lett.

[CR61] Zhuang X, Papathomas TV (2009). Prior entry for feature-based attention: are objects of the attended color perceived earlier. J Vis.

